# Association of sleep and generalized anxiety disorder in Korean adolescents

**DOI:** 10.1186/s12889-024-19524-4

**Published:** 2024-07-30

**Authors:** Ah Jung Ko, Jinhyun Kim, Eun-Cheol Park

**Affiliations:** 1 Department of Health Policy & Management, Graduate School of Public Health, Yonsei University, Seoul, Republic of Korea; 2 Institute of Health Services Research, Yonsei University, Seoul, Republic of Korea; 3 Department of Preventive Medicine, Yonsei University College of Medicine, 50-1 Yonsei-Ro, Seodaemun-Gu, Seoul, 03722 Republic of Korea; 4 Department of Psychiatry, Yonsei University Hospital, Seoul, Republic of Korea

**Keywords:** Anxiety, Sleep, Social jet lag, Differences in sleep duration between weekdays and weekends, KYRBS

## Abstract

**Background:**

Generalized anxiety disorder (GAD) is a common anxiety disorder among adolescents, significantly impacting their concentration and learning capabilities. The connection between emotional well-being and sleep is well-established, and Korean adolescents are particularly prone to inadequate sleep. This study aimed to determine the association between sleep duration and GAD in Korean adolescents.

**Methods:**

This study was conducted using data from 106,513 adolescents aged 12–18 years. Data from the 2020–2022 Korea Youth Risk Behavior Survey were used. Sleep duration was classified into five groups, based on an average sleep duration of 7–7.9 h in adolescents. Social jet lag was defined as a misalignment between an individual's biological and social clocks. Differences in sleep duration between weekdays and weekends, social jet lag, and bedtime were each classified into three categories. Multiple logistic regression analysis was performed to evaluate the association between sleep duration and GAD.

**Results:**

Comparing the five groups classified based on sleep duration, adolescents in the groups that slept less experienced a significant increase in the odds of developing GAD (adjusted odds ratio [aOR]: boys: 1.10 in the 6.0–6.9-h group, 1.14 in the 5.0–5.9-h group, and 1.23 in the ≤ 4.9-h group; girls: 1.05 in the 6.0–6.9-h group, 1.19 in the 5.0–5.9-h group, 1.22 in the ≤ 4.9-h group). Adolescents with poor sleep quality experienced more frequent instances of inadequate sleep (aOR: boys: 2.51; girls: 2.43).

**Conclusions:**

GAD is strongly associated with insufficient sleep. Consequently, it is imperative to assess and address GAD in adolescents with irregular sleep patterns.

## Background

Adolescence is the transitional phase characterized by both biological and emotional changes. As a result, adolescents become more susceptible to internal issues [1]. Mental disorders have a significant impact on the overall life and function of adolescents and can affect them in the long run until adulthood [2]. Generalized anxiety disorder (GAD) is common among adolescents with mental disorders [3]. The lifetime prevalence of GAD in adolescents is 3% [4]. However, anxiety disorders, such as GAD, are difficult to diagnose and are often not appropriately treated [5]. Previous studies have shown that patients with GAD have more comorbidities and worse quality of life than patients without GAD. Furthermore, the comorbidities increase and quality of life worsens significantly as the severity of GAD increases, resulting in occupational disorders and an increased cost of life for patients with GAD [6]. GAD in adolescents is highly related to the morbidity rate [7]. Individuals with GAD suffer functional damage to their bodies and roles, require more days to recover from diseases, suffer more work loss, and require higher use of medical services [8]. In the case of adolescents with GAD, attention should be paid to their parental attachment. Unstable parental attachment contributes to the development of GAD symptoms in adolescents [9]. Adolescents with GAD are characterized by excessive concern about various problems, including death and social, school, and family problems [10]. Anxiety and the belief that there will be problems are key factors in adolescent GAD [10, 11]. Adolescent GAD increases negative emotions and fatigue and lowers positive emotions. In adolescents, GAD not only causes comorbid diseases but also affects lifestyle habits [12]. Anxiety in adolescence is associated with harmful drinking habits, smoking impulses, and fewer leisure activities [12, 13].

Adequate and consistent sleep, along with exercise and proper nutrition, significantly impact the mental and physical health of adolescents [14]. Therefore, sleep is an important health indicator of adolescents [15]. Prolonged sleep deprivation can result in cognitive and mental health issues, as well as chronic fatigue. Sleep plays an important role in adolescents’ overall performance, general health, development [16], and mental health in many ways [17]. The occurrence and continuation of sleep problems are associated with the diagnosis of anxiety disorders. Inadequate sleep exacerbates mental health disorders [18]. Adolescents' inadequate and irregular sleep patterns are a common problem around the world [19]. Despite evidence showing that sleep deprivation is more prevalent in adolescents than in other age groups, many countries fail to acknowledge or address this issue adequately. If sleep deprivation persists, it can cause cognitive and mental health problems as well as fatigue [14]. Asian adolescents, in particular, tend to have shorter sleep durations, with some getting 1–2-h lesser sleep than their European counterparts [20, 21]. As adolescents grow older, they tend to face pressure to reduce their sleep duration due to academic reasons. On average, adolescents sleep for 7.4 h [22]. Among Korean adolescents, middle school students typically sleep for an average of 7.2 h and high school students for 5.8 h [23].

Moreover, adolescents often struggle to maintain consistent sleep patterns for an entire week due to academic commitments. Hormonal changes caused by these irregular sleep patterns have led to the assumption that they can adversely affect mental health [24]. Irregular sleep patterns are associated with poor mental health and behavioral disorders [25]. Differences in sleep duration between weekdays and weekends are related to health outcomes, such as concentration, life satisfaction, and emotional and physical symptoms. The reported difference in sleep duration between weekdays and weekends among Korean teenagers is 96–133 min [26].

According to previous research, sleep patterns tend to align with natural phenomena [27]. This is referred to as the biological clock [28]. However, in modern times, with the advancement of science and technology enabling the creation of artificial light, human sleep times have become aligned with social schedules [28]. This has led to changes and differences in sleep patterns, which in turn have negative impacts on health [28].

The inconsistency in sleep patterns between workdays and rest days is known as social jet lag. Social jet lag is known to reduce mental health, attention, and academic achievements [29]. Nonetheless, previous studies have struggled to establish consistent findings regarding the relationship between social jet lag and mental health [30, 31]. Furthermore, late bedtime is significantly associated with an increased prevalence of depression and anxiety in adolescents. Previous studies have shown that the later adolescents go to sleep, the stronger this association [32, 33].

Several sleep-related variables have been found to affect mental health. This study aimed to quantify the impact of sleep duration on pan-anxiety disorders by comparing various variables, offering insights into opportunities for the prevention and intervention of GAD.

## Methods

### Study population

The Korea Youth Risk Behavior Survey (KYRBS) is conducted annually by the Korea Centers for Disease Control and Prevention and the Ministry of Education to understand the health behaviors of middle and high school teenagers across the country. The KYRBS is an anonymous self-reported online survey in which students from 400 middle and 400 high schools were selected using a two-stage cluster sampling method. In the first sampling step, schools were categorized as stratification variables representing regional groups and school levels, and the sample schools were selected by random sampling. During the second sampling period, one class was randomly extracted from each grade [34]. The study utilized 16th–18th (2020–2022) KYRBS that was conducted on 161 and 646 students in middle school and high school, respectively [35]. Participants who reported sleep durations exceeding 24 h and those with missing data were excluded. Before participating in the survey, the purpose and procedure of the survey were explained to the students and they gave their consent to participate online [33].

### Measurements

The dependent variable was the GAD-7, which is the optimal standard used for assessing GAD. The GAD-7 is a self-report questionnaire used to quickly identify GAD in primary care patients [36]. GAD-7 comprises seven questions regarding the previous 2-week period. The seven questions are regarding the following: tension and anxiety, inability to stop or control worry, being too worried, difficulty in resting, restlessness, being easily annoyed or afraid [37]. The GAD-7 classifies the responses to the questions as “completely ignored,” “interrupted for several days,” “disturbed for more than 7 d,” and “disturbed almost every day,” giving them 0, 1, 2, and 3 points, respectively. The scores of all responses are combined to evaluate the degree of normal, minor, secondary, and serious anxiety disorders according to the criteria in the GAD-7 [37]. GAD was assessed as normal, mild, moderate, and serious when the GAD-7 score was ≤ 4, 5–9, 10–14, and ≥ 15 points, respectively [36]. In this study, we defined the presence of GAD in the participants as a GAD-7 score ≥ 5 points [38]. The Cronbach's α coefficient for the GAD-7 was 0.900.

The primary independent variable was a sleep duration, which was subdivided into five groups. This variable was derived from questionnaires regarding the sleep duration of the participant in the past week. The classification of sleep duration was based on the average sleep time of adolescents, and was classified into ≤ 4.9, 5.0–5.9, 6.0–6.9, 7.0–7.9 and ≥ 8.0 h based on the average sleep duration of 7.0–7.9 h in adolescents [39].

The covariates included sleep patterns (social jet lag, bedtime, differences in sleep duration between weekdays and weekends, and sleep quality), demographic factors (sex and age), and socioeconomic and health-related factors (income, region, academic score, alcohol consumption status, smoking status, physical activity, perceived stress level, and self-reported health status).

The corrected social jet lag was used, which is the absolute value of the difference between the sleep start time on the working day and the sleep start time on the day off. A difference < 1.0 h was classified as no social jet lag, 1.0–1.9 h as low social jet lag, and ≥ 2.0 h as high social jet lag [29, 40]. The quality of sleep was based on the participants' subjective evaluation and was classified as good, normal, or poor. Bedtime was defined as the average sleep start time and was classified as < 24:00, 24:00–02:00, and ≥ 2:00. Total difference in sleep duration between weekdays and weekends was classified as < 1.0 h, 1.0–1.9 and ≥ 2.0 h. Sex was classified as female and male, and socioeconomic status, academic score, perceived stress level and self-reported health status were all classified as low, middle, and high. Age was classified into 12–15 and 16–18 years. The area of residence was classified as a metropolitan city, small- or medium-sized city, or county, based on the size of the city. Smoking and alcohol consumption statuses were defined as “ever” or “never.”

### Statistical analysis

A multiple logistic regression model was used to investigate the association between sleep duration and GAD. We conducted the analysis after stratifying the participants by sex. We used Statistical Analysis System version 9.4 (SAS Institute Inc., Cary, NC, USA) for all analyses, and statistical significance was set at a p-value of < 0.05. The results were presented as adjusted odds ratios (aORs) with corresponding 95% confidence intervals (CI). A multinomial logistic regression analysis was performed to determine the relationship between sleep duration (≤ 4.9, 5.0–5.9, 6.0–6.9, 7.0–7.9 and ≥ 8.0 h) and the likelihood of mild, moderate, and severe GAD. Subgroup analyses were conducted based on the bedtime (< 24:00, 24:00–02:00, and ≥ 2:00), sleep quality (good, normal, or poor), social jet lag (no, low, and high) and difference in sleep duration between weekdays and weekends (< 1.0 h, 1–1.9 h, and ≥ 2 h).

## Results

Table 1 shows the results of the multiple regression analysis between independent variables, including sleep duration, stratified by sex and the prevalence of GAD. Of the 106,513 participants, 54,829 were boys and 51,684 were girls. The prevalence of GAD was 27.9% in boys (*n* = 15,215) and 20.1% in girls (*n* = 21,296).

**Table 1:** Socioeconomic and health-related characteristics of all participants according to generalized anxiety disorder

Variables		Male				*p*-value	Female				*p*-value
		No GAD		GAD			No GAD		GAD		
		N	(%)	N	(%)		N	(%)	N	(%)	
Total(*N* = 106,513)		39,614	(72.8)	15,215	(27.9)		30,388	(59.1)	21,296	(20.1)	
**Sleep duration(hours)**						< .0001					< .0001
	-4.9	6,069	(66.4)	3,071	(33.6)		5,727	(50.7)	5,561	(49.3)	
	5.0–5.9	5,483	(65.9)	2,835	(34.1)		5,623	(53.9)	4,808	(46.1)	
	6.0–6.9	9,276	(70.7)	3,845	(29.3)		7,569	(59.5)	5,161	(40.5)	
	7.0–7.9	9,582	(75.4)	3,133	(24.6)		6,278	(64.5)	3,456	(35.5)	
	8.0-	9,204	(79.8)	2,331	(20.2)		5,191	(69.2)	2,310	(30.8)	
**Social jet lag**						0.1191					< .0001
	No	25,763	(72.3)	9,865	(27.7)		19,857	(59.9)	13,303	(40.1)	
	Low	7,837	(72.7)	2,941	(27.3)		6,196	(57.8)	4,527	(42.2)	
	High	6,014	(71.4)	2,409	(28.6)		4,335	(55.6)	3,466	(44.4)	
**Differences in sleep duration between weekdays and weekends(hours)**						< 0.0001					< 0.0001
	-0.9	10,402	(74.3)	3,597	(25.7)		6,149	(60.7)	3,981	(39.3)	
	1.0–1.9	8,933	(73.0)	3,310	(27.0)		6,641	(60.3)	4,380	(39.7)	
	2.0-	13,754	(70.7)	5,701	(29.3)		13,254	(58.3)	9,466	(41.7)	
**Sleep quality**						< .0001					< .0001
	Poor	11,110	(59.5)	7,577	(40.5)		11,492	(47.9)	12,495	(52.1)	
	Normal	14,084	(74.4)	4,835	(25.6)		10,634	(64.3)	5,910	(35.7)	
	Good	14,420	(83.7)	2,803	(16.3)		8,262	(74.1)	2,891	(25.9)	
**Age**						< .0001					< .0001
	12–15	23,131	(73.9)	8,156	(26.1)		17,994	(60.4)	11,777	(39.6)	
	16–18	16,377	(70.0)	7,010	(30.0)		12,349	(56.6)	9,467	(43.4)	
**Income**						< .0001					< .0001
	Low	4,184	(61.7)	2,593	(38.3)		2,827	(45.0)	3,459	(55.0)	
	Middle	18,581	(73.0)	6,887	(27.0)		15,736	(59.5)	10,698	(40.5)	
	High	16,849	(74.6)	5,735	(25.4)		11,825	(62.4)	7,139	(37.6)	
**Academic score**						< .0001					< .0001
	Low	12,416	(69.1)	5,547	(30.9)		8,799	(53.0)	7,808	(47.0)	
	Middle	12,067	(74.4)	4,159	(25.6)		9,955	(61.3)	6,298	(38.7)	
	High	15,131	(73.3)	5,509	(26.7)		11,634	(61.8)	7,190	(38.2)	
**Alcohol status**						< .0001					< .0001
	No	25,647	(74.9)	8,605	(25.1)		23,037	(62.6)	13,751	(37.4)	
	Yes	13,967	(67.9)	6,610	(32.1)		7,351	(49.3)	7,545	(50.7)	
**Smoking status**						< .0001					< .0001
	No	34,809	(73.3)	12,679	(26.7)		28,958	(60.0)	19,336	(40.0)	
	Yes	4,805	(65.5)	2,536	(34.5)		1,430	(42.2)	1,960	(57.8)	
**Physical activity**						0.0001					0.0169
	No	4,364	(70.2)	1,852	(29.8)		8,636	(58.0)	6,258	(42.0)	
	Yes	35,250	(72.5)	13,363	(27.5)		21,752	(59.1)	15,038	(40.9)	
**Perceived stress level**						< .0001					< .0001
	Low	13,195	(94.6)	757	(5.4)		6,976	(92.8)	545	(7.2)	
	Middle	19,115	(78.4)	5,267	(21.6)		15,979	(73.4)	5,790	(26.6)	
	High	7,304	(44.3)	9,191	(55.7)		7,433	(33.2)	14,961	(66.8)	
**Self-reported health status**						< .0001					< .0001
	Low	1,771	(45.6)	2,111	(54.4)		1,568	(30.8)	3,520	(69.2)	
	Middle	7,060	(61.7)	4,374	(38.3)		7,152	(49.3)	7,367	(50.7)	
	High	30,783	(77.9)	8,730	(22.1)		21,668	(67.5)	10,409	(32.5)	
**Region**						< .0001					< .0001
	Metropolitan city	17,622	(73.3)	6,421	(26.7)		13,308	(60.2)	8,800	(39.8)	
	Small & mid. City	18,890	(71.3)	7,607	(28.7)		14,869	(57.9)	10,825	(42.1)	
	county	3,102	(72.3)	1,187	(27.7)		2,211	(57.0)	1,671	(43.0)	
**Depression**						< .0001					< .0001
	No	34,824	(80.5)	8,409	(19.5)		25,599	(72.2)	9,870	(27.8)	
	Yes	4,790	(41.3)	6,806	(58.7)		4,789	(29.5)	11,426	(70.5)	

*Abbreviation*: *GAD* generalized anxiety disorder

Table 2 shows the associations between sleep duration and other factors. In boys, the likelihood of developing GAD was significantly increased in the groups with sleep duration < 7.0 h compared with the group with sleep duration of 7.0–7.9 h. (< 5.0-h group: aOR, 1.23; 95% CI, 1.11–1.39, 5.0–5.9-h group: aOR, 1.14; 95% CI, 1.05–1.24, 6.0–6.9-h group: aOR, 1.1; 95% CI, 1.02–1.18). In girls, the likelihood of GAD significantly increased in the groups with sleep duration < 6.0 h compared with the group with sleep duration of 7–7.9 h. (< 4.9 h group: aOR, 1.22; 95% CI, 1.11–1.35, 5.0–5.9-h group: aOR, 1.19; 95% CI, 1.10–1.29).

**Table 2:** Results of logistic Regression analysis to investigate the association between sleep pattern and generalized anxiety disorder

Variables		Generalized Anxiety Disorder (GAD-7)							
		Male				Female			
		aOR		95%CI		aOR		95%CI	
**Sleep duration(hours)**									
-4.9		1.23	(1.11	-	1.39)	1.22	(1.11	-	1.35)
5.0–5.9		1.14	(1.05	-	1.24)	1.19	(1.10	-	1.29)
6.0–6.9		1.10	(1.02	-	1.18)	1.05	(0.98	-	1.14)
7.0–7.9		1.00				1.00			
8.0-		0.93	(0.86	-	1.01)	0.97	(0.90	-	1.07)
**Social jet lag**									
No		1.00				1.00			
Low		0.93	(0.88	-	1.00)	1.04	(0.98	-	1.11)
High		0.96	(0.90	-	1.04)	1.14	(1.06	-	1.24)
**Differences in sleep duration between weekdays and weekends(hours)**									
-0.9		1.00				1.00			
1.0–1.9		1.07	(1.00	-	1.15)	0.96	(0.90	-	1.04)
2.0-		1.05	(0.98	-	1.14)	0.88	(0.82	-	0.96)
**Sleep quality**									
Good		1.00				1.00			
Normal		1.65	(1.53	-	1.79)	1.42	(1.33	-	1.54)
Poor		1.25	(1.17	-	1.35)	1.09	(1.01	-	1.19)
**Age**									
12–15		1.00				1.00			
16–18		0.92	(0.88	-	0.99)	0.87	(0.83	-	0.93)
**Income**									
Low		1.17	(1.08	-	1.28)	1.3	(1.20	-	1.42)
Middle		0.99	(0.94	-	1.06)	1.03	(0.98	-	1.10)
High		1.00				1.00			
**Academic score**									
Low		1.00				1.00			
Middle		0.92	(0.86	-	0.99)	0.97	(0.92	-	1.04)
High		0.95	(0.89	-	1.02)	1.08	(1.01	-	1.15)
**Alcohol status**									
No		1.00				1.00			
Yes		1.07	(1.01	-	1.14)	1.19	(1.12	-	1.27)
**Smoking status**									
No		1.00				1.00			
Yes		0.98	(0.91	-	1.07)	1.07	(0.96	-	1.21)
**Physical activity**									
No		1.00				1.00			
Yes		0.97	(0.90	-	1.06)	0.89	(0.85	-	0.95)
**Perceived stress level**									
Low		1.00				1.00			
Middle		3.78	(3.44	-	4.17)	3.69	(3.32	-	4.11)
High		12.04	(10.86	-	13.37)	13.64	(12.25	-	15.20)
**Self-reported health status**									
Low		1.59	(1.50	-	1.69)	1.49	(1.41	-	1.58)
Middle		2.36	(2.15	-	2.61)	2.12	(1.93	-	2.33)
High		1.00				1.00			
**Region**									
Metropolitan city		1.00				1.00			
Small & mid. City		1.08	(1.02	-	1.15)	1.06	(1.01	-	1.13)
County		1.11	(0.98	-	1.27)	1.06	(0.97	-	1.17)
**Depression**									
Yes		1.00				1.00			
No		3.28	(3.09	-	3.49)	3.39	(3.22	-	3.58)

*Abbreviation*: *OR* adjusted odds ratio, *CI* confidence interval

Table 3 shows the results of the subgroup analyses based on the total difference in sleep duration between weekdays and weekends, social jet lag, bedtime, and sleep quality compared across groups varying in average sleep duration. The longer the sleep duration, the lower were the odds of developing GAD within all subgroups, stratified by bedtime, quality of sleep, social jet lag, and total difference in sleep duration between weekdays and weekends. Particularly, in the group with 1 h difference in sleep duration between weekdays and weekends, the difference in GAD likelihood between the ≤ 4.9 h (boys: aOR, 2.21; 95% CI, 1.91–2.57 and girls: aOR, 2.24; 95% CI, 1.97–0.57) and ≥ 8-h (boys: aOR, 0.69; 95% CI, 0.63–0.76 and girls: aOR, 0.68; 95% CI, 0.61–0.77) groups was the largest. In the poor-quality sleep group, the longer the sleep duration, the lower was the likelihood of GAD among both boys and girls.

**Table 3:** Subgroup analysis based on sleep pattern

Variables		Generalized Anxiety Disorder (GAD-7)																			
		< 5 h				5-6 h				6-7 h				7-8 h			> 8 h				
		aOR		95%CI		aOR		95%CI		aOR		95%CI		**aOR **		aOR	**95%CI **				
**Male**																					
**Differences in sleep duration between weekdays and weekends(hours)**	-0.9	2.21	(1.91	-	2.57)	1.47	(1.32	-	1.65)	1.22	(1.11	-	1.34)	1.00	0.69		(0.63	-	0.76)		
	1.0–1.9	2.13	(1.83	-	2.49)	1.37	(1.23	-	1.54)	1.33	(1.22	-	1.46)	1.00	0.76		(0.69	-	0.85)		
	2.0-	2.13	(1.87	-	2.43)	1.71	(1.58	-	1.87)	1.28	(1.20	-	1.38)	1.00	0.93		(0.85	-	1.02)		
**Bedtime**	-23.59	2.41	(0.41	-	14.12)	1.27	(0.67	-	2.40)	1.19	(1.04	-	1.38)	1.00	0.70		(0.65	-	0.76)		
	24:00–01:59	1.87	(1.41	-	2.49)	1.39	(1.25	-	1.56)	1.28	(1.19	-	1.39)	1.00	0.92		(0.78	-	1.11)		
	02:00-	2.10	(1.92	-	2.30)	1.60	(1.49	-	1.73)	1.26	(1.16	-	1.37)	1.00	1.24		(0.83	-	1.89)		
**Social jet lag**	No	1.52	(1.43	-	1.64)	1.54	(1.44	-	1.66)	1.26	(1.19	-	1.36)	1.00	0.74		(0.69	-	0.81)		
	Low	2.15	(1.79	-	2.59)	1.45	(1.29	-	1.65)	1.27	(1.16	-	1.40)	1.00	0.72		(0.64	-	0.82)		
	High	2.00	(1.60	-	2.51)	1.93	(1.67	-	2.24)	1.16	(1.05	-	1.30)	1.00	1.00		(0.89	-	1.13)		
**Sleep quality**	Good	0.76	(0.68	-	0.86)	0.77	(0.66	-	0.90)	0.65	(0.59	-	0.73)	1.00	0.50		(0.46	-	0.55)		
	Normal	1.15	(1.04	-	1.28)	1.29	(1.17	-	1.44)	1.05	(0.97	-	1.14)	1.00	0.90		(0.82	-	0.99)		
	Poor	2.51	(2.33	-	2.72)	2.07	(1.92	-	2.25)	2.00	(1.86	-	2.15)	1.00	1.87		(1.67	-	2.10)		
**Female**																					
**Differences in sleep duration between weekdays and weekends(hours)**	-0.9	2.24	(1.97	-	2.57)	1.45	(1.31	-	1.62)	1.19	(1.08	-	1.31)	1.00	0.68		(0.61	-	0.77)		
	1.0–1.9	1.90	(1.67	-	2.17)	1.64	(1.49	-	1.82)	1.17	(1.07	-	1.29)	1.00	0.75		(0.67	-	0.86)		
	2.0-	2.03	(1.84	-	2.25)	1.57	(1.47	-	1.69)	1.23	(1.15	-	1.32)	1.00	0.93		(0.85	-	1.02)		
**Bedtime**	-23.59	1.43	(0.25	-	8.14)	1.56	(0.87	-	2.81)	1.07	(0.92	-	1.26)	1.00	0.73		(0.67	-	0.80)		
	24:00–01:59	1.65	(1.32	-	2.07)	1.33	(1.21	-	1.48)	1.1	(1.01	-	1.20)	1.00	0.79		(0.66	-	0.97)		
	02:00-	2.04	(1.89	-	2.21)	1.59	(1.49	-	1.71)	1.32	(1.23	-	1.43)	1.00	1.52		(1.03	-	2.27)		
**Social jet lag**	No	1.66	(1.56	-	1.77)	1.43	(1.33	-	1.54)	1.11	(1.04	-	1.20)	1.00	0.72		(0.66	-	0.79)		
	Low	2.47	(2.17	-	2.83)	1.75	(1.59	-	1.93)	1.25	(1.15	-	1.37)	1.00	0.84		(0.74	-	0.96)		
	High	2.49	(2.07	-	3.01)	2.03	(1.77	-	2.34)	1.54	(1.39	-	1.72)	1.00	1.08		(0.96	-	1.22)		
**Sleep quality**	Good	0.76	(0.67	-	0.87)	0.89	(0.78	-	1.03)	0.67	(0.61	-	0.75)	1.00	0.52		(0.48	-	0.58)		
	Normal	1.21	(1.11	-	1.34)	1.15	(1.05	-	1.27)	0.93	(0.86	-	1.02)	1.00	0.87		(0.79	-	0.96)		
	Poor	2.43	(2.28	-	2.61)	1.95	(1.82	-	2.10)	1.78	(1.65	-	1.92)	1.00	1.57		(1.40	-	1.76)		

*Abbreviation*: *aOR* adjusted odds ratio, *CI* confidence interval

Table 4 shows the results of multinomial logistic regression analysis of the association between sleep duration and mild, moderate, and severe GAD. Both boys and girls showed significant association with severe (boys: aOR, 151; 95% CI, 1.25–1.85 and girls: aOR, 1.66; 95% CI, 1.42–1.96), moderate (boys: aOR, 1.26; 95% CI, 1.10–1.46 and girls: aOR, 1.32; 95% CI, 1.18–1.49), and mild (boys: aOR, 1.08; 95% CI, 1.00–1.17 and girls: aOR, 1.15; 95% CI, 1.07–1.25) GAD in the group with sleep duration ≤ 4.9 h.

**Table 4:** Results of the Multinomial logistic regression analysis of the associations between sleep duration and mild, moderate, severe GAD (generalized anxiety disorder)

Variables		GAD Mild (5–9)				GAD Moderate (10–14)				GAD Severe (15-)			
		aOR		95%CI		aOR		95%CI		aOR		95%CI	
**Sleep duration**													
** Male**													
-4.9		1.08	(1.00	-	1.17)	1.26	(1.10	-	1.46)	1.51	(1.25	-	1.85)
5.0–5.9		1.11	(1.02	-	1.22)	1.21	(1.06	-	1.41)	1.15	(0.94	-	1.43)
6.0–6.9		1.08	(1.01	-	1.17)	1.06	(0.94	-	1.21)	1.02	(0.84	-	1.24
7.0–7.9		1.00				1.00				1.00			
8.0-		0.94	(0.87	-	1.03)	0.94	(0.81	-	1.09)	1.00	(0.80	-	1.25)
** Female**													
-4.9		1.15	(1.07	-	1.25)	1.32	(1.18	-	1.49)	1.66	(1.42	-	1.96)
5.0–5.9		1.17	(1.08	-	1.28)	1.14	(1.02	-	1.30)	1.21	(1.03	-	1.44)
6.0–6.9		1.06	(0.98	-	1.15)	1.04	(0.93	-	1.16)	1.07	(0.91	-	1.26)
7.0–7.9		1.00				1.00				1.00			
8.0-		0.97	(0.89	-	1.06)	0.94	(0.82	-	1.09)	1.00	(0.80	-	1.25)

## Discussion

In this study, we found that GAD was associated with sleep duration and sleep patterns in Korean adolescents. The association between inadequate sleep and GAD is consistent with the results of previous studies [41–43]. In other studies, GAD showed sex differences in adolescents, with girls having a higher prevalence and cumulative hazard of anxiety disorders compared with boys [44, 45]. In our study, girls showed a higher rate of mild GAD according to the GAD-7. Less than 20% of adolescents in this study slept for an average of ≥ 8 h a day, just as adolescents do not sleep sufficiently globally.

By subdividing insufficient sleep duration into further categories, we focused on the occurrence of anxiety according to sleep duration. We found that, based on an average sleep duration of 7.0–7.9 h, the shorter the sleep time, the higher is the probability of GAD occurrence, regardless of sex. In the group with sleep duration ≥ 8 h, the probability of developing GAD decreased; however, this decrease was not significant.

Previous studies have shown that late bedtimes and differences between sleep durations on weekdays and weekends are linked to emotional instability, substance abuse, smoking, and suicidal thoughts [46]. Abnormal sleep patterns affect adolescents' mental pain as well as their health behavior [46]. Sleep time coincides with night time, which naturally occurs because of the rotation of the earth. However, in modern times, the sleep time in humans is altered by artificial lighting, and social lag is a change in sleep patterns due to the difference between the individual’s original biological clock and the social clock. Social jet lag has been found to be associated with chronic sleep deprivation, and consequently cognitive performance, mood disorders, and attention deficit hyperactivity disorders [27]. In another study, differences in sleep duration between weekdays and weekends had a greater effect on mental health than on physical health. Reducing the difference in sleep duration between weekdays and weekends can be an important factor in improving mental health in adolescents because a greater difference in sleep duration between weekdays and weekends is associated with aggression and decreased concentration [26]. Previous studies have suggested sleep as an interventional tool in the cognitive behavioral therapy process, because lower sleep quality is significantly associated with GAD and depression [47].

As shown in Table 3, in each subgroup, the longer the sleep duration, the lower were the odds of developing GAD. Regarding the total difference in sleep duration between weekdays and weekends, in the group with a 0.0–0.9-h difference, the odds of developing GAD lowered from 2.24 to 0.68 among girls when the sleep duration increased from < 5 h to ≥ 8 h. In terms of social jet lag, the odds of developing GAD increased in girls in the groups of no, low, and high social jet lag (in that order); the higher the sleep duration, the lower were the odds of developing anxiety disorders. This means that social jet lag is associated with the GAD and can be reduced by sleep time intervention. A similar trend was observed between sleep quality and the odds of developing GAD. In each subgroup, overall, shorter sleep duration significantly increased the likelihood of developing GAD. However, if the quality of sleep was poor, the odds of developing GAD was significantly higher even if the sleep duration was long.

This study has some limitations. First, this was a cross-sectional study using data collected at specific time points; as cross-sectional data lack a time dimension, it was not possible to establish causal relationships, despite the observed associations. Second, because the data were collected through a self-reported web-based survey, they might not be accurate because of the possibility of recall bias or participants misunderstanding the questions. Third, since the survey was conducted among teenagers aged 12–18 years, the questionnaire was intentionally simplified. Despite the limitations, this study may be generalized to adolescents in Korea because it is based on nationwide data.

## Conclusions

This study revealed that various sleep-related variables in Korean adolescents are associated with GAD. However, if the requirement of adequate sleep duration is satisfied, the odds of developing GAD are lowered. Therefore, sufficient sleep time is an important factor among the many sleep variables associated with GAD. This also suggests that sleep duration may serve as a potential indicator of untreated and undiagnosed GAD among adolescents, offering opportunities for the prevention and intervention of mental disorders. Therefore, educational efforts are needed to help adolescents recognize the importance of adequate sleep.

## Data Availability

No datasets were generated or analysed during the current study.

## Abbreviations

GAD: Generalized anxiety disorder
KYRBS: Korea Youth Risk Behavior Survey
aOR: Adjusted odds ratio
CI: Confidence interval
